# Genetic characterization and phylogenetic study of Indonesian cuscuses from Maluku and Papua Island based on *16S rRNA* gene

**DOI:** 10.14202/vetworld.2020.2319-2325

**Published:** 2020-11-04

**Authors:** Rini Widayanti, Richo Apriladi Bagas Pradana, Rony Marsyal Kunda, Suhendra Pakpahan

**Affiliations:** 1 Department of Biochemistry and Molecular Biology, Faculty of Veterinary Medicine, Universitas Gadjah Mada, Yogyakarta, Indonesia; 2 Biology Study Program, Faculty of Mathematics and Natural Sciences, Universitas Pattimura, Ambon, Indonesia; 3 Research Center for Biology, Indonesian Institute of Sciences (LIPI), Cibinong, West Java, Indonesia

**Keywords:** Indonesian cuscuses, Maluku, Papua, *Phalanger*, phylogeny, *Spilocuscus*

## Abstract

**Background and Aim:**

Indonesian cuscuses are now becoming scarce because of the reduction of habitat and poaching. Further, molecular characterization of Indonesian cuscuses is still very lacking. This study aimed to determine genetic markers and phylogenetic relationships of Indonesian cuscuses based on *16S rRNA* gene sequences.

**Materials and Methods:**

This study used 21 cuscuses caught from two provinces and 16 islands: 13 from Maluku and eight from Papua. Cuscus samples were taken by biopsy following ethics guidelines for animals. The genome isolation was done using gSYNC DNA Mini Kit (Geneaid Biotech Ltd., Taiwan). The *16S rRN*A gene was amplified by primers (16SKUSAF and 16SKUSAR), and the polymerase chain reaction product obtained was 1875 base pair (bp). The analysis of genetic characterization and the phylogenetic relationship was performed usingMEGA version X software (https://www.megasoftware.net/).

**Results:**

*16S rRNA* gene sequencing attained 1598 bp for all samples. Based on the *16S rRNA* nucleotide sequences, cuscuses from Papua and Maluku belong to the genus *Phalanger* and *Spilocuscus*. *Phalanger* spp. and *Spilocuscus* spp. from Papua can be distinguished from *Phalanger* and *Spilocuscus* from Maluku, except *Spilocuscus* from Ternate has a very close relationship with cuscus from Sentani, Papua.

**Conclusion:**

Indonesian cuscuses were derived into two clades based on *16S rRNA* gene sequence, one group to genus *Phalanger* and another group to *Spilocuscus*.

## Introduction

Animal distribution studies are increasingly being conducted, so the characterization of each species needs to be carried out both morphologically and molecularly. One animal originating from the Wallace line is the small Sulawesi cuscus (*Strigocuscus celebensis*); another is Sulawesi bear cuscus (*Ailurops ursinus*). There are at least 20 additional species which belong to the family Phalangeridae [1-3]. *A. ursinus* is spread throughout Sulawesi and nearby islands in Togian, Peleng, Muna, Buton, and Lirung (Talaud Islands). *Spilocuscus maculatus*, described by Desmarest [4], has a range that extends from Queensland (Australia), through Papua, to Buru and Caram, and the small island of Selayar, at the tip of the Southeast Sulawesi. Peninsula cuscus is an Australian animal, marsupial mammal, belonging to the family Phalangeridae, whose distribution is limited in East Indonesia (Sulawesi, Maluku, and Papua), Australia, and Papua New Guinea. From the five genus cuscus, four genera are found in Indonesia, namely, *Ailurops*, *Phalanger*, *Spilocuscus*, and Strigocuscus, and there are at least 24 species of cuscus spread across the three islands [5-7]. In Papua, two genera were found, namely, *Phalanger* (bottled cuscus) and *Spilocuscus* (spotted cuscus); in Maluku, there are two genera found, namely, *Phalanger* and *Spilocuscus*; and in Sulawesi, the genus *Spilocuscus* and *Ailurops* are found, which are endemic species in Sulawesi Island [8-10]. This research is significant because Indonesian cuscuses are now beginning to become scarce due to the decreasing habitat it occupies, many local people are hunting for consumption, trade, and, in some communities, ritual purposes [11,12].

Cuscus is one of the protected wildlife in Indonesia based on the decree of the Indonesian Ministry of Agriculture No. 247/KPTS/UM4/1979 and PP. No. 7 of 1999 concerning Preservation of Plants and Animals. Efforts to maintain the preservation of these animals need to be done *in situ* and *ex situ* conservation. The data from these animals, both morphologically and genetically, are lacking, so the molecular characteristics need to be studied more closely. Latinis [13] has researched hunting cuscus in Central Maluku. Fatem and Sawen [14] identified cuscus in the northern coastal region of Manokwari, Papua, based on its morphological characteristics. Molecular research on cuscus has been carried out by Munemasa *et al*. [15] using the mitochondrial genome but at the level of the Phalangeridae family. Molecular studies at the species level have never been carried out. According to the previous studies, the NADH dehydrogenase subunit 3 (ND3) gene sequence, the 4L NADH dehydrogenase subunit (ND4L) gene, and 12 rRNA gene can be used as genetic markers of *Tarsius bancanus* from Sumatra, Kalimantan with Tarsius from Sulawesi [16-18].

This study aimed to obtain nucleotide sequences and measure diversity using *16S rRNA* mitochondrial gene in cuscus from Maluku and Papua. These mitochondrial DNA sequences are unique and can be used as genetic markers for species identification. The *16S rRNA* gene has been widely used for phylogenetic study and detection in other species [19-22]. It is expected that the nucleotide diversity of each cuscus species can be used as genetic markers and can determine the phylogenetic relationship of the cuscuses from Maluku and Papua. It is hoped that by identifying genetic markers will help conservation efforts, especially for animals that have lost their habitat and need to be conducted *ex situ*. The information gained from these efforts can apply to other endangered species that are threatened with extinction, preserving the biological wealth in Indonesia.

## Materials and Methods

### Ethical approval

This study was approved by the Animal Ethics Committee for using Animal and Scientific Procedures in the Faculty of Veterinary Medicine, Universitas Gadjah Mada, Indonesia.

### Sample collection, study period, and location

**Figure-1:**
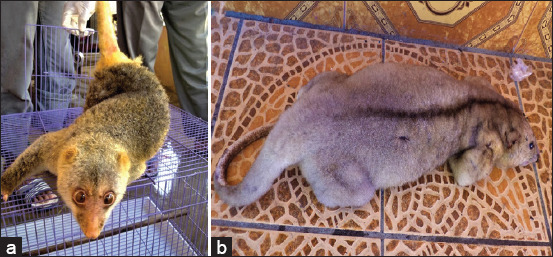
The cuscuses were from Maluku (a) and Papua (b).

Cuscus samples were taken from their natural habitat, namely, Maluku Province (13 individuals) and Papua Province (8 individuals) (Table-1). All cuscus samples were identified based on morphological characteristics (Figure-1) and sample tissues were preserved in RNA latter buffer (Qiagen, Germany). The Indonesian cuscus samples in this study were unrelated genetically because they were taken individually from the habitat of each location. This research was conducted from January to June 2020, starting from sample collection to data analysis.

**Table-1:** Origin of cuscus samples from Maluku and Papua Island.

Origin of cuscus	
Maluku Island	Papua Island
Allang P. Ambon	Moor Nabire Island
Geser Island	Nabire Island
Gorom Island	Sentani Jayapura Island
Halmahera Island	Wanggar Nabire Island
Hatu Ambon Island	Yaro Nabire Island
Kariu Haruku Island	
Lakor Island	
Manipa Island	
North Seram Island	
South Seram Island	
Soya Ambon Island	

### Genome isolation

Total DNA was extracted from tissue biopsy (30 mg). Isolation and purification of DNA were performed using a DNA Isolation Kit (Qiagen). The isolated DNA was detected after migration on a 1% agarose gel using a 1 × TBE buffer in the Submarine Electrophoresis device (Hoefer, USA). We observed the isolates with the help of ultraviolet (UV) light (λ = 260 nm) after the gel was stained with Bioatlas (Genaid, Taiwan). The isolated DNA was then stored at −20°C before being used for the next step.

### Primer design

The primers are designed using the Primer3 output program (http://www-genome.wi.mit.edu/cgi.bin/primr3.cgi/results_from-primer3) based on the mitochondrial sequences of *Phalanger vestitus* (Access number AB241057.1). The sequence of the primers for the amplification of the *16S rRNA* gene is presented in Table-2.

**Table-2:** Primer sequences for amplifying *16S rRNA* gene.

Primer	Sequence	Product size (bp)	Tm (°C)
16SKUSAF	5’ TTAGGAAGGCA	1875	51
	ATTGCTAGG 3’		
16SKUSAR	5’ CCGTCACCC		53
	TCCTCAATTA 3’		
16SKUSBF	5’ TTAGAAAAGCA	1875	54
	ATTGCTAGG 3’		
16SKUSBR	5’ CGTCACCCTC		
	CTCAATTA 3’	53	

### Amplification of the 16S *rRNA* gene by polymerase chain reaction (PCR)

Genomic DNA was used as DNA template for the amplification of *16S rRNA* gene. DNA amplification by PCR in this study used an Infinigen PCR machine. Amplification of the *16S rRNA* gene each using a self-designed pair of primers based on *P. vestitus* mitochondrial genome sequence from GenBank (access number AB241057.1), as shown in Table-1. DNA amplification was carried out under the following conditions: Initial denaturation for 2 min at temperature 94°C then followed by 94°C for 30 s for denaturation, 49°C for 45 s for primary attachment (annealing), and 72°C for 1 min 45 s for elongation; amplification was carried out as many as 35 cycles, then ending with 5 min at 72°C.

PCR products were detected by 1.5% agarose gel using a 1 × TBE buffer in the Submarine Electrophoresis device (Hoefer, USA). The observation was carried out on UV transilluminator (λ = 260 nm) after the gel was stained with Bioatlas (Genaid). DNA markers with a size of 1000 bp are used as indicators of molecular weight.

### DNA sequencing

The amplified PCR product was purified using the GFX Column purification kit, then used as DNA template for sequencing. For each sample, two sequencing reactions were carried out using the forward and reverse primers. Sequencing was performed on all *16S rRNA* gene PCR products that good sequencing results were marked with a single peak spectrophotogram graph and no noise.

### Amplification of 16S *rRNA* gene by PCR technique

**Figure-2:**
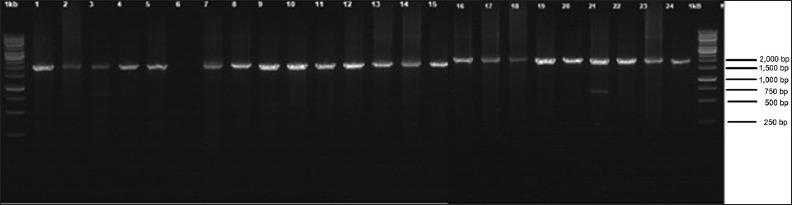
Polymerase chain reaction product of *16S rRNA* cuscus from Papua and Maluku in agarose gel 1%.

Genome amplification of 21 cuscus samples from Papua and Maluku resulted in excellent bands, with a length of approximately 1875 bp. Amplicons were visualized using 1% agarose gel electrophoresis with 1 kb DNA ladder as a molecular weight indicator (Figure-2). The PCR products were sequenced using forward primers and reverse primers used for the amplification process.

### Statistical analysis

The multiple alignments of *16S rRNA* gene nucleotides were analyzed using ClustalW software [23]. The sequences of *16S rRNA* gene were analyzed by MEGA software version 7 [24]. Genetic distance was analyzed by the Kimura method with two parameters [25]. PCR products were sequenced, both forward and reverse. Sequencing results were aligned using the ClustalW program. Reverse sequences were used as comparisons of forward sequences to obtain an accurate sequence result. The phylogenetic tree was constructed based on nucleotide sequences by the neighbor-joining method with a bootstrap value 1000×. The cuscus used as a comparison and was taken from GenBank data including *P. vestitus* (Access number AB241057.1), *S. maculatus* (KJ868160.1), *Phalanger gymnotis* (KJ868142.1), *Phalanger orientalis* (AY228381.1), and *Trichosurus vulpecula* (AF357238.1).

## Results and Discussion

### Genetic variation

**Figure-3:**
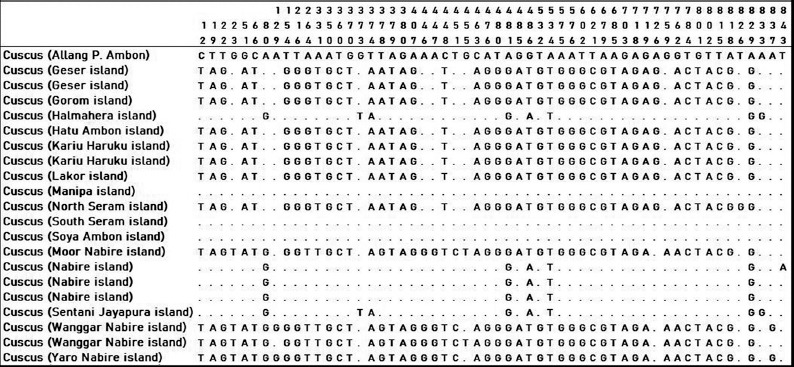
Variable sites 12-843 on 16S gene of cuscus from Maluku and Papua.

**Figure-4:**
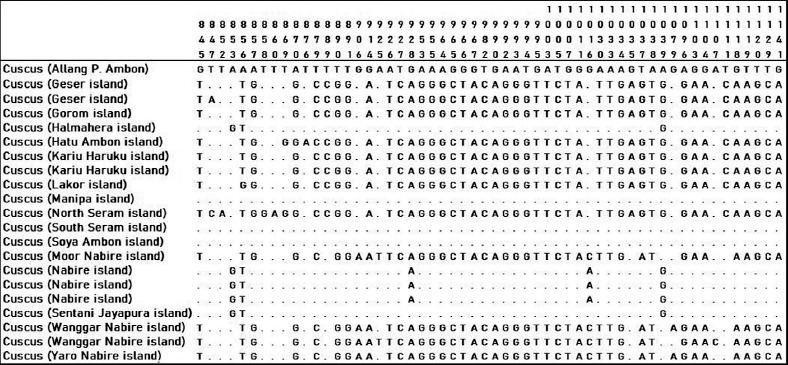
Variable sites 845-1141 on 16S gene of cuscus from Maluku and Papua.

**Figure-5:**
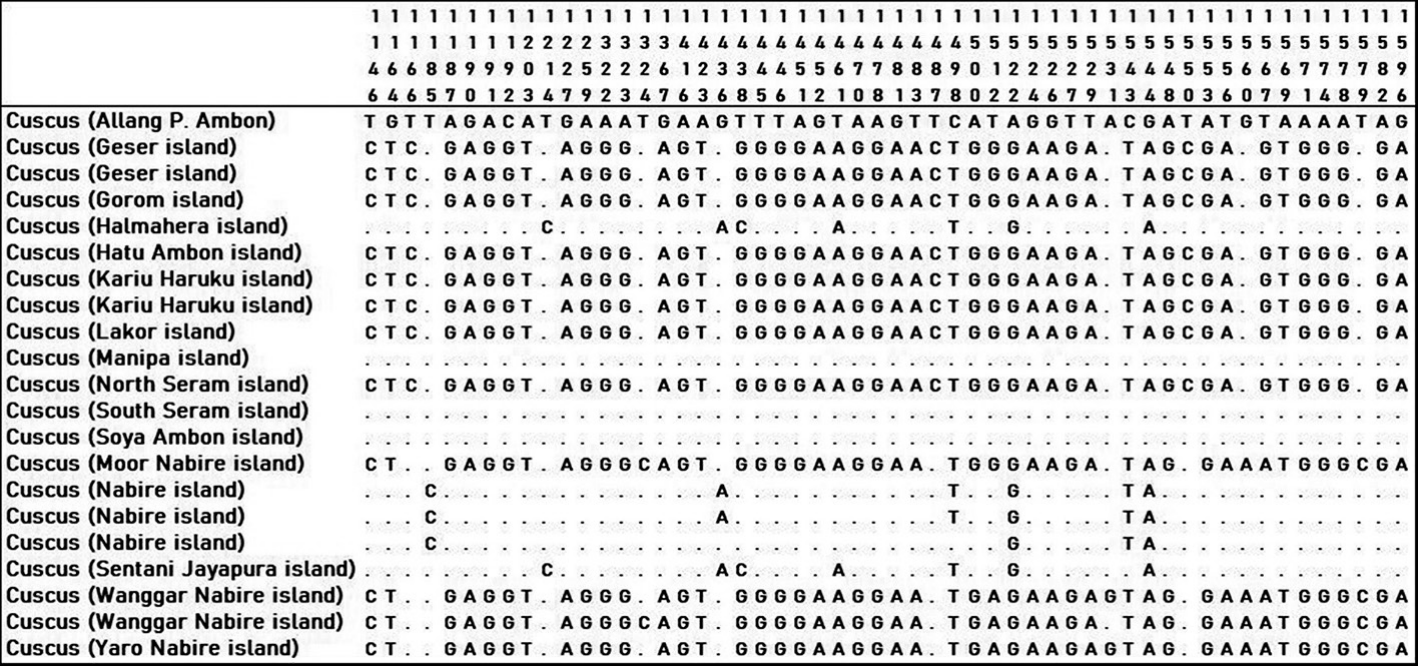
Variable sites 1146-1596 on 16S gene of cuscus from Maluku and Papua.

Twenty-one cuscus samples from Papua and Maluku were analyzed based on sequences of forward and reverse *16S rRN*A sequences. Forward and reverse sequences were used for the editing process by observing sequence electropherogram. Cuscus (Allang P. Ambon) was a basis comparison to determine the site variables for other cuscuses. The dot on the alignment image showed homology with cuscus (Allang P. Ambon). There were 166 site variables in the amplified 16S gene (Figures 3-5). Some cuscus samples had high similarity with cuscus (Allang P. Ambon), namely, cuscus Halmahera, Manipa, South Seram, Soya Ambon, and Nabire Island.

The alignment sequence showed that there were two groups of cuscuses in this study which had similarities in the 16S gene sequence. The first group is cuscus (Allang P. Ambon, Halmahera, Manipa, South Seram, Soya Ambon, and Nabire), the second group is cuscus (Geser, Gorom, Hatu Ambon, Kariu Haruku, Lakor, North Seram, Moor Nabire, Wanggar Nabire, and Yaro Nabire). This separation of groups did not follow their area of origin cuscuses.

### Genetic relationship between cuscus from Maluku and Papua

**Figure-6:**
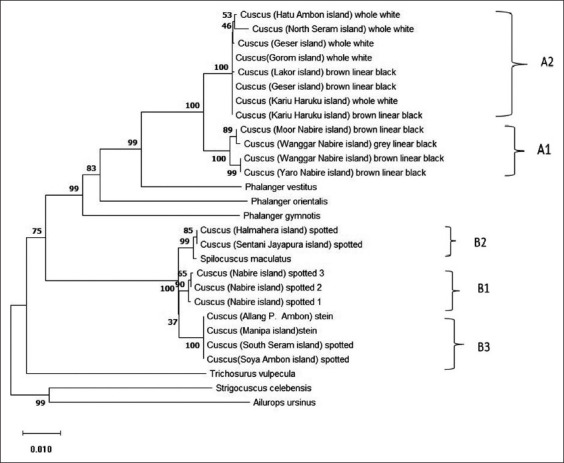
Evolutionary relationships of Indonesian cuscuses (with color of coat) and some cuscuses from GenBank database *Phalanger vestitus* (Access number AB241057.1), *Spilocuscus maculatus* (KJ868160.1), *Phalanger gymnotis* (KJ868142.1), *Phalanger orientalis* (AY228381.1), and *Trichosurus vulpecula* (AF357238.1).

The phylogenetic tree was constructed using the neighbor-joining method. Cuscuses from Maluku and Papua were divided into two clades (Figure-6), which means that cuscuses were split into two genera. Clade A1 was the cuscuses from Papua belonging to the genus *Phalanger*, while clade A2 was the cuscuses from Maluku belonging to the genus *Phalanger*. Clade B1 was the cuscuses from Papua belonging to the genus *Spilocuscus*, while clade B3 was the cuscuses from Maluku belonging to the genus *Spilocuscus*. In the B2 clade, the cuscuses came from Papua and Maluku.

The number of base substitutions per site from averaging over all sequence pairs between groups is shown (Table-3). Standard error estimates are shown above the diagonal. Analyses were conducted using the maximum composite likelihood model [1]. All positions containing gaps and missing data were eliminated. There were 1543 positions in the final dataset.

**Table-3:** Estimates of evolutionary divergence over sequence pairs between groups.

Cuscus	*S. macalatus*	*Phalanger*	Cuscus from Ambon	Cuscus from Papua
S. macalatus*		0.008	0.006	0.004
Phalanger*	0.091		0.006	0.006
Cuscus from Maluku	0.063	0.076		0.005
Cuscus from Papua	0.045	0.081	0.054	

* From GenBank data, *Spilocuscus maculatus* (KJ868160.1), *Phalanger vestitus* (access number AB241057.1)

### Evolutional history

The evolutionary history was inferred using the neighbor-joining method [26]. The optimal tree with the sum of branch length = 0.40235225 is shown. The percentage of replicate trees in which the associated taxa clustered together in the bootstrap test (1000 replicates) is shown next to the branches [27]. The tree is drawn to scale, with branch lengths in the same units as those of the evolutionary distances used to infer the phylogenetic tree. The evolutionary distances were computed using the Tamura-Nei method [28] and are in the units of the number of base substitutions per site. This analysis involved 28 nucleotide sequences, both the samples of current study and samples from GenBank. All ambiguous positions were removed for each sequence pair (pairwise deletion option). There were 1598 positions in the final dataset. In Figure-2, the cuscuses from Papua and Maluku belong to two genera, namely, *Phalanger* and *Spilocuscus*. In the group of *Phalanger*, those from Papua and Maluku had the closest relationship with *P. vestitus*, while *Spilocuscus* type had the closest relationship with *S. maculatus*. Two species of cuscus are commonly hunted in Maluku, *S. maculatus* and *P. orientalis. S. maculatus* is known as the spotted cuscus. The distribution of *S. maculatus* includes New Guinea, Aru, Kei, Buru, Seram, Ambon, Selayer, Banda, Pandjang, Timor, and Cape York Peninsula, Australia. Prehistory and archeology of the cuscus evidenced that *P. orientalis* may have been actively introduced to Maluku, Seram, Buru, Sanana, and the Kai Islands as early as 6500 years ago. *P. vestitus* and *S. maculatus* were found in Papua and Maluku region [13,29]. The recent study on cuscus from Maluku, Kusumaningrum, and Abinawanto [30] studied cuscuses based on COI gene sequences and found that there were two types of cuscus, namely, *S. maculatus* and *P. vestitus*.

The results showed that, for the cuscus from Maluku and Papua, each origin included two genera, namely, the genus *Phalanger* and *Spilocuscus*. Widayanti *et al*. [8] studied molecular characterization of cuscus from Maluku and Papua Island based on the NADH dehydrogenase subunit 1 gene, grouping cuscus from Papua and Maluku became two genera *Phalanger* and *Spilocuscus* and classified into *Phalanger* spp. and *S*. *maculatus* species. The taxonomic review based on the biogeographic history of Phalangeridae is complicated because the geological history of Southeast Asia is very complicated to describe. As a result, the historical interpretation of phalangerids biogeography provided can only be temporary [1-3]. The molecular phylogeny presented in this study is thought to reflect better the evolutionary relationships of Phalangeridae that differ from previous taxonomic determinations; the molecular phylogeny tree shows high suitability using statistical calculations [31].

Cuscuses of Papua and Maluku belonging to the genus *Phalanger* (Figure-6) showed that the two cuscus groups are separated in different sub-branches and show that *Phalanger* from Papua and Maluku can be distinguished [8,9]. Usmany *et al*. [11] reported that four types of cuscus lived in Seram (Maluku), namely, brown cuscus (*P. orientalis*), gray cuscus (*P. vestitus*), white cuscus (*Phalanger ursinus*), and spotted cuscus (*S. maculatus*). These four types of cuscus showed similarities in morphology. The spotted cuscus and gray cuscus have similarities of the earlobe, which are covered with hair. Still, white cuscus and brown cuscus are similar in that the earlobe is not covered with hair [14]. Likewise, for Papua and Maluku cuscuses belonging to the genus *Spilocuscus* are also in different sub-branches. However, except for Tobelo (ternate) cuscus, the cuscus has historically been brought along with the migration of people from Papua to Maluku (Ternate) so that cuscus from Ternate has a very close relationship with those from P. Sentani, Papua [13,29].

## Conclusion

Cuscuses from Papua and Maluku based on the *16S rRNA* gene nucleotide sequence are classified in the genus *Phalanger* and *Spilocuscus*. *Phalanger* spp. and *Spilocuscus* from Papua can be distinguished from *Phalanger* and *Spilocuscus* from Maluku, except *Spilocuscus* from Ternate has the closest relationship with cuscus from Sentani, Papua. The *16S rRNA* gene can be used to identify genetic markers and differentiate cuscus species from Maluku and Papua. Genetic characterization information, especially genetic markers, is needed to carry out the conservation and breeding of each species.

## Authors’ Contributions

RW and SP designed this research and collected cuscus samples for this study. SP, RABP, and RMK conducted research in the laboratory. RW and SP analyzed the data and wrote the manuscript. All authors have read and approved the final manuscript.
